# Colopleural fistula caused by aspergillus: an extremely rare complication after lung resection—case report

**DOI:** 10.1186/s40792-016-0167-0

**Published:** 2016-04-23

**Authors:** Akio Hayashi, Yoshiyuki Susaki, Naoko Ose, Yukiyasu Takeuchi, Hajime Maeda

**Affiliations:** Department of Thoracic Surgery, National Hospital Organization, Toneyama National Hospital, 5-1-1, Toneyama, Toyonaka, Osaka 560-8552 Japan

**Keywords:** Lung infection, Fistula (colopleural), Surgery complications

## Abstract

A colopleural fistula is a rare condition reported to be caused by Crohn’s disease, a malignant tumor of the gastrointestinal tract, and other clinical conditions. Some studies have noted that a sub-diaphragmatic abscess, usually organized following abdominal surgery, may play some role in the formation of this type of fistula. Therefore, a colopleural fistula is a complication very rarely encountered by thoracic surgeons after lung resection.

We experienced an extremely rare case of colopleural fistula following a left lower lobectomy for lung aspergillosis. Here, we report a 71-year-old man with a surgical history of proximal gastrectomy for gastric cancer. He underwent left lower lobectomy of the lung for aspergillosis, and a colopleural fistula occurred on the second operative day as a complication. Aspergillus might be responsible for forming a fistula between the colon and lung via the diaphragm, and lung surgery manifested this rare condition. Although some reports suggest that surgical treatment is mandatory to cure this fistula, an immediate colostomy in our case reduced the internal pressure of the colon, thus enabling spontaneous closure of the fistula with appropriate drainage and antibiotics. The patient was discharged in a good condition.

## Background

Colopleural fistula as a complication after lung resection is a quite rare condition. Few cases of colopleural or colobronchial fistula have been reported, and those fistulas are caused by Crohn’s disease, malignant tumor of gastrointestinal tract, or other clinical conditions. On the other hand, the aspergillus species are sometimes detected in the lung in immune-compromised hosts and are liable to infect preexisting destroyed lung. Aspergillus invades by destroying lung structure and causes hemosputum, hemoptysis, and other severe complications. Here, we describe an extremely rare case with colopleural fistula caused by lung aspergillus which was revealed after lung resection. The patient had gotten in serious straits but was successfully treated with immediate colostomy and appropriate drainage.

## Case presentation

A 71-year-old man had hypertension and diabetes mellitus and had been regularly examined by a home doctor. An abnormal lung shadow was first detected in 2009, but bronchoscope examination revealed no bacteria, fungi, or malignant cells. He consulted the home doctor complaining of hemosputum and fever in 2012. His chest X-ray and blood test revealed that he is suffering lung aspergillosis. Oral antimycotic medicine was administered, but his symptoms prolonged. The patient was referred to our institution for surgical resection in 2012 because focus of the infection was localized in the left lower lobe.

**Fig. 1:**
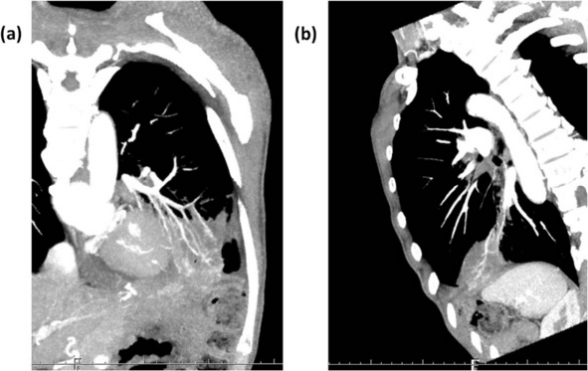
Preoperative chest CT of **a** coronal view and **b** sagittal view showed an irregular mass in the left lower lung, but no distinct fistula was detected

He had a history of proximal gastrectomy because of gastric cancer 9 years ago. He suffered from hypertension and diabetes mellitus. He had been a one-pack-a-day smoker for 30 years, and he stopped smoking 20 years ago. When he was referred to our hospital, his body mass index was 17.9 with cough and without fever up (36.7 °C). Respiratory sounds were diminished on the left lower side. Laboratory findings on admission were as follows: leukocyte 8610/mm^3^, C-reactive protein 0.6 mg/dl, hemoglobin 9.9 g/dl, glucose 183 mg/dl, hemoglobin A1c 6.7 %, β-D-glucan 26.5 pg/dl, and serum aspergillus antibody positive. Cultivation test of sputum detected *Escherichia coli*, *Enterobacter aerogenes*, and *Enterococcus faecium*. Spirometry showed that his respiratory functions were kept normal. Chest radiography showed localized consolidation on the left lower side of the lung. Chest computed tomography (CT) showed an irregular mass which contacts widely to the diaphragm, but distinct fistula was not detected (Fig. 1).

**Fig. 2:**
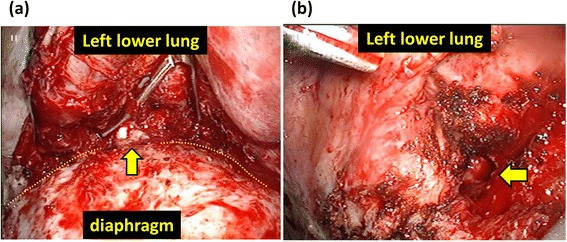
Operative views with VATS. The *dot line* indicates severe adhesion between the lung and diaphragm. White pus flew out of a cavity (*arrows*) while dissecting the adhesion

**Fig. 3:**
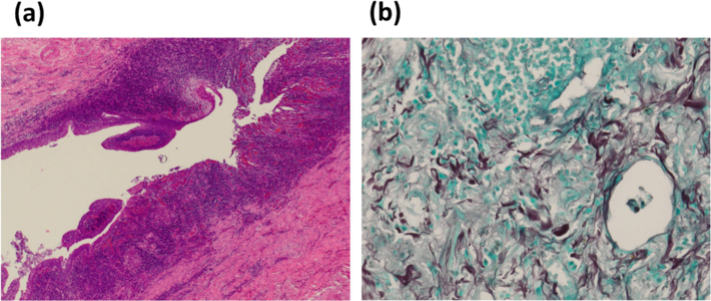
Pathological findings of the resected lung stained with **a** hematoxylin and eosin and **b** Grocott methenamine silver detected fungus body in the tissue adjacent to the fistula

Video-assisted thoracic surgical left lower lobectomy was performed under general anesthesia with thoracic epidural anesthesia using a 4-cm utility incision and three ports. The lower lobe adhered severely to the diaphragm, of which the anterior basal segment (S8) was most prominent. During the resection of the adhesion, white pus, not feces, flew out of a cavity beside the diaphragm (Fig. 2). Close observation revealed that the cavity was neither inside of the lung parenchyma nor of the intestinal tract. The localized peri-diaphragmatic abscess cavity was widely opened, and the thoracic cavity was well laved during the operation. A chest tube was placed just beside the cavity and apex of the pleural cavity. Blood loss was 425 g, and operative time was 378 min. The Pathological findings of tissue sampled during the operation (HE staining and Grocott staining) showed fungus body (Fig. 3). Culture inspection of the resected tissue adjacent to the cavity detected *E. faecium*.

**Fig. 4:**
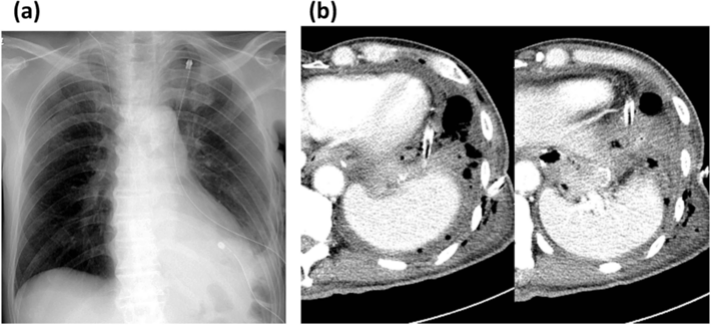
**a** Chest X-ray showed good expansion of the remaining left lung. **b** Chest computed tomography showed fluid collection around the chest drainage tube right above the diaphragm

**Fig. 5:**
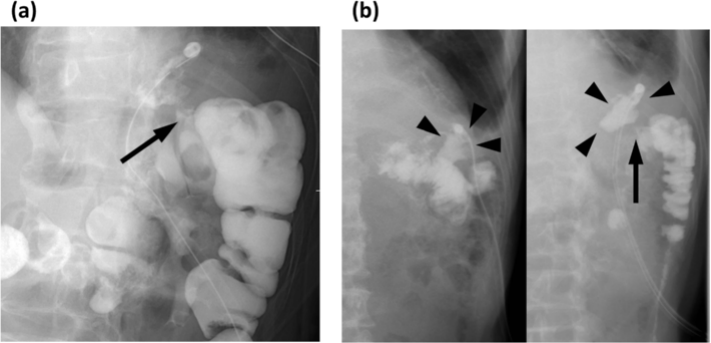
**a** Contrast enema on the 3rd postoperative day showed a colopleural fistula (*arrow*). **b** Fistulography on the 25th postoperative day also showed the fistula (*arrow*), but the cavity was small and localized in the lower pleural space (*arrow heads*)

On the 2nd postoperative day (POD), the lower chest tube drained feculent fluid. Chest CT showed low density area around the chest tube right above the diaphragm (Fig. 4), but not pneumoperitoneum. Fluid collected in the lower pleural cavity, but the upper pleural cavity was filled with well-expanded lung without any air space. The patient was immediately transferred to a gastrointestinal surgery department of an adolescent institution under the diagnosis of colopleural fistula and received colostomy on the following day. Oral intake was started on the 6th POD, and the patient was transferred back to our institute on the 16th POD. Contrast enema was performed on the 3rd POD and fistulography on the 25th POD; both revealed communication between the splenic flexure of the colon and left pleural cavity, but contrast medium remained in the small localized cavity (Fig. 5). Considering these findings and the patient’s condition, we continued conservative treatment to control the infection. The chest tube was cut shorter on the 25th POD and was removed on the 50th POD as the cavity had shrunk gradually. The patient was discharged on the 63rd POD in a good condition.

### Discussion

Aspergillus is an infectious fungal disease that occurs most often in the lungs of people with suppressed immune system and is one of the most popular lung mycoses in the field of thoracic surgery. However, colopleural fistula and colobronchial fistula are very rare pathological conditions, especially for thoracic surgeons. Some reports have claimed that the sub-diaphragmatic abscess may play some role in the formation of these fistulas. Though it is an old report, Ochsner et al. summarized 3608 sub-diaphragmatic abscesses and colobronchial fistula and pyothorax arose in 10.5 and 17.8 % of them, respectively [1]. On the other hand, intra-peritoneal abscess occurs in 0.3–5.3 % after surgery for gastric cancer, and the sub-diaphragmatic abscess is the most common [2]. In this presenting report, our patient had undergone a major surgical intervention below the left diaphragm for gastric cancer 9 years ago. This surgical procedure may be the cause of the sub-diaphragmatic abscess, and communication between the colon and the abscess cavity might have already existed prior to thoracic surgery this time. Findings of preoperative sputum culture also indicated the existing fistula.

Aspergillus produces some protease, fibrinase, and various toxins, and they destroy the lung structure which sometimes causes severe complications [3, 4]. This is a very rare case that destructive invasion of lung aspergillus caused fistula through the diaphragm, although whether inflammation which caused adhesion of the lung and diaphragm is due to aspergillus or sub-diaphragmatic abscess is unclear. Review of the literature revealed that these rare complications, colopleural or colobronchial fistula, are reported to be caused by malignant tumor, Crohn’s disease, and other clinical conditions [5–8]. However, to the best of our knowledge, this is the first report which indicated the relevance of aspergillus to the formation of fistulous communication between the pleural cavity and adjacent organs below the diaphragm.

**Fig. 6:**
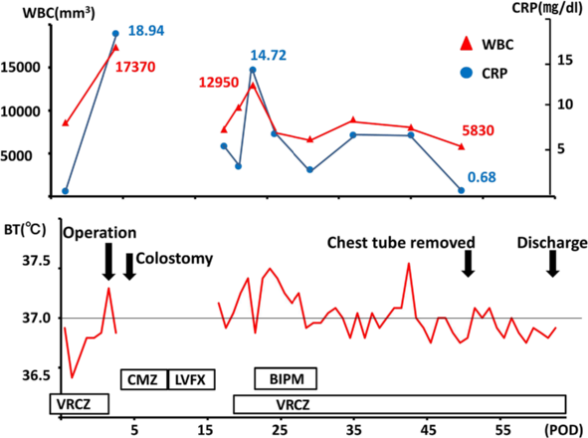
The figure indicates the time course of the patient’s laboratory data, body temperature, and details of treatments. The patient was generally in good condition during the treatment

As for the treatment of this medical condition, conservative therapy was adopted in this case while previous reports suggested that surgical treatment is mandatory [8]. First, surgical resection and closure of the fistula might induce the patient great stress because of the probable adhesion in his left epigastric region due to gastric surgery. Second, the chest tube was properly placed to drain feculent discharge. Actually, the infectious cavity was limited in the lower pleural cavity. We also confirmed that the lower pleural cavity was separated on the 17th POD when the chest tube of the pulmonary apex was removed: indocyanine green reagent was injected from the apical chest tube, but green discharge was not drained from lower chest tube. Third, the patient had been in good condition all along without high fever (Fig. 6). Considering all these findings, intra-thoracic infection was controlled with conservative therapy.

## Conclusions

We experienced a rare and life-threatening condition, colopleural fistula, which was actualized after surgical lung resection for aspergillosis. Although the patient had diabetes mellitus, immediate colostomy reduced internal pressure of the colon and enabled spontaneous closure of fistula with appropriate drainage and antibiotics.

### Consent

Written informed consent was obtained from the patient’s family for publication of this case report and any accompanying images. A copy of the written consent is available for review by the Editor-in-Chief of this journal.

## Abbreviations

CT: computed tomography
POD: postoperative day
